# Exploring the Impact of Diabetes on Kidney Transplant: Patient Outcomes and Management Strategies

**DOI:** 10.7759/cureus.80843

**Published:** 2025-03-19

**Authors:** Hiba Hatim H Eltayeb, Akash Rawat, Juan Felipe Salazar González, Farah N Ahmad, Jaime T Lee Young, Farah Algitagi, Lintha Z Khattak, Ikhlas U Qazi, Abhya Arya, Zummar F Asad, Iqrah A Issimdar, Humza F Siddiqui

**Affiliations:** 1 Medicine, University of Georgia, Tbilisi, GEO; 2 General Medicine, Himalayan Institute of Medical Sciences, Swami Rama Himalayan University, Dehradun, IND; 3 General Medicine, Clínica Renovar, Villavicencio, COL; 4 Family Medicine, Dubai Health, Dubai, ARE; 5 Surgery, Port of Spain General Hospital, Port of Spain, TTO; 6 Medicine, Hashemite University, Zarqa, JOR; 7 Surgery, Khyber Medical College, Peshawar, PAK; 8 General Surgery, King’s College London, London, GBR; 9 Emergency, Deen Dayal Upadhyay Hospital, New Delhi, IND; 10 Medicine and Surgery, Royal College of Surgeons in Ireland, Dubai, ARE; 11 Health and Agriculture, University College Dublin, Dublin, IRL; 12 Internal Medicine, Jinnah Postgraduate Medical Centre, Karachi, PAK

**Keywords:** diabetic kidney disease (dkd), glucose-lowering medications, immunosuppression, kidney transplantation (kt), kidney transplant recipients (ktrs), new-onset diabetes after transplantation (nodat), simultaneous pancreas-kidney transplantation (spkt)

## Abstract

Diabetic kidney disease (DKD) is a serious consequence of diabetes mellitus (DM). If not managed effectively, DKD often develops into end-stage renal disease (ESRD). The most successful treatment for ESRD is kidney transplantation, offering improved quality of life and survival rates. For insulin-dependent diabetic patients with ESRD, simultaneous pancreas-kidney transplantation (SPKT) offers a treatment alternative that treats both kidney failure and the underlying diabetes. However, SPKT involves more complicated surgery, prolonged operative time, and a higher risk of complications. This review aims to highlight the impact of DM on kidney transplant recipients (KTRs) regarding post-transplant complications, graft survival, mortality rates, and the role of glucose-lowering medications and immunosuppressants. The incidence of urinary tract infections, cardiovascular complications, and diabetic foot disease was higher among KTRs. A decrease in graft survival rate at five years was observed among diabetics compared to non-diabetics, with similar graft survival rates among type 1 and type 2 DM. The mortality rate was notably higher among diabetic patients, with cardiovascular complications being the leading cause. The emergence of new-onset diabetes mellitus post-transplantation (NODAT) is a significant cause of concern. Certain risk factors, including a family history of DM, age >45 years, obesity, male gender, and immunosuppressive medications, have been linked to this phenomenon. Immunosuppression is a substantial challenge among diabetics as certain medications such as tacrolimus have shown to be considerably diabetogenic compared to cyclosporine and belatacept, and it is also postulated that corticosteroids can lead to hyperglycemia. Some studies proved that glucose-lowering medications, including insulin degludec, glucagon-like peptide-1 receptor agonists, thiazolidinediones, and sodium-glucose cotransporter 2 inhibitors, are safe and effective among KTRs. However, these studies are debatable and of low confidence. Hence, it is imperative to conduct large clinical trials and establish definitive guidelines to manage pre-existing diabetes and NODAT among KTRs with multidisciplinary care to help clinicians improve patient outcomes.

## Introduction and background

Diabetes mellitus (DM) is one of the most prevalent metabolic disorders characterized by hyperglycemia. Type 1 diabetes (T1D) occurs due to insulin deficiency and is more common in children with an incidence rate of less than 10%. On the other hand, type 2 diabetes (T2D) occurs due to insulin resistance accounting for approximately 90% of the cases [1,2]. This condition can lead to serious complications including diabetic kidney disease (DKD), which primarily results in declining kidney function [3]. According to estimates from the World Health Organization (WHO), DM is projected to rank among the top 10 leading causes of death globally by 2030 [1,2]. Although the prevalence of DKD among diabetics has not altered over the last decades, the rising worldwide incidence of DM suggests an increased risk of developing this condition [4]. The prevalence of DKD among T1D patients is far less than in patients with T2D [5]. If inadequately managed, DM and diabetic nephropathy (DN) can accelerate the onset of chronic kidney disease (CKD), which has a global prevalence typically ranging from 11% to 13% [6]. Moreover, CKD diagnosis is made after three months of structural and functional kidney impairment. The Kidney Disease Improving Global Outcomes (KDIGO) staging system classifies CKD into five main stages depending on the estimated glomerular filtration rate (eGFR) and urinary albumin levels [7]. Over time, in 30%-50% of the cases, uncontrolled diabetes and CKD may progress to end-stage renal disease (ESRD), which is defined by the KDIGO staging as an eGFR of less than 15 mL/minute [8,9].

Over the past few decades, advancements in managing DM and DN have significantly improved patient outcomes [5,10]. Patient awareness and encouraging lifestyle modifications are the cornerstones of the care plan [11]. Medications are tailored to the individual’s preferences and symptoms to achieve certain objectives, such as protecting vital organs from failing, reducing the number of complications and risk factors, and, ultimately, hindering the progression to ESRD [3,5]. Renal replacement therapy (RRT) serves as the first-line treatment for patients with ESRD. It encompasses organ transplantation, hemodialysis, and peritoneal dialysis. Moreover, dialysis may be initiated as a short-term option before transplantation or as a definitive therapy for patients who are not eligible for surgery, depending on their preferences and clinical symptoms [6,11-14].

**Figure 1:**
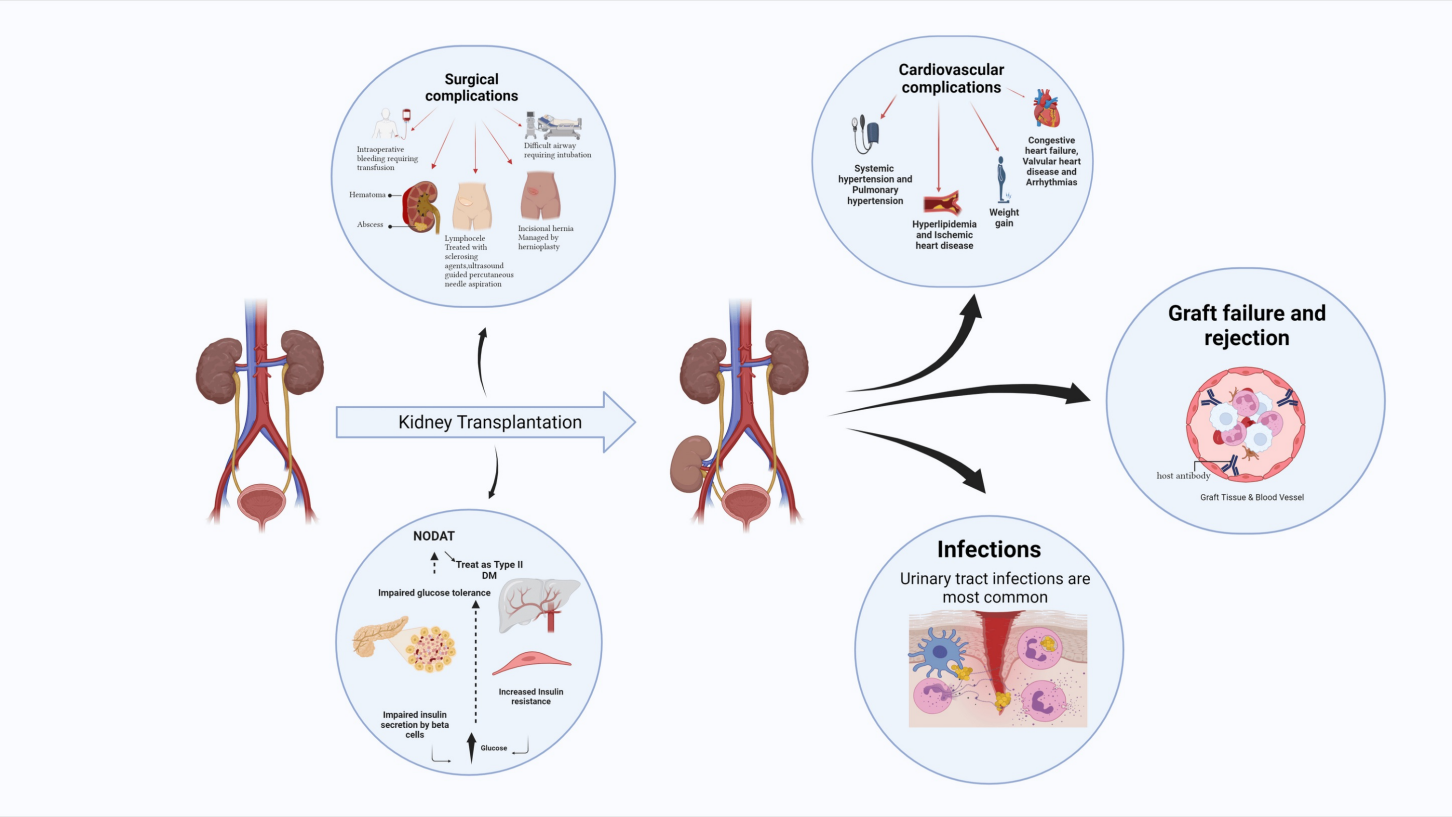
Complications and outcomes of kidney transplantation in correlation to diabetes mellitus. Figure made using biorender.com. NODAT: new-onset diabetes after transplantation; DM: diabetes mellitus

One viable treatment approach is kidney transplantation (KT), which improves the patient’s quality of life and increases their survival rate, as individuals with DKD have a higher risk of cardiovascular complications. This procedure can be performed alone or in conjunction with pancreas transplantation, which not only mitigates the advancement of diabetes-induced organ damage but also restores normal glucose levels. However, only 10% of individuals in need are likely to get an allograft due to the low availability of donor organs [6,11,15]. Post-transplant diabetes mellitus (PTDM) is a common complication affecting many individuals following KT. New-onset diabetes after transplantation (NODAT) is associated with several detrimental effects on both patient and graft survival. Furthermore, the administration of post-transplant immunotherapy is linked to the development of NODAT. During the first year after KT, the incidence of this condition ranges between 10% and 20%, and it increases over time [15,16]. Nevertheless, due to the high death rate linked to cardiovascular disorders, physicians continue to face significant challenges in managing DKD and DN, despite the emergence of several therapeutic approaches worldwide [7] (Figure 1).

This narrative review is a comprehensive resource that aims to provide a thorough understanding of the current evidence behind KT for DKD management. It explores various aspects, including the indications and contraindications of the transplant, different surgical approaches and techniques, and their impact on patients’ outcomes. It also provides a detailed description of how diabetes should be managed after the transplant procedure and a brief review of new-onset PTDM in previously non-diabetic patients, as well as current challenges and future directions of this therapeutic approach.

## Review

Kidney and pancreas transplant

Indications

Currently, the best treatment option for ESRD is KT. However, a kidney transplant does not address the most common cause of end-stage renal failure, T2D. The leading cause of mortality in T1D is also ESRD, causing over 50% of deaths before the age of 35 years [17]. By performing a simultaneous kidney-pancreas transplant, both ESRD that was a consequence of T2D can be effectively treated. A simultaneous pancreas-kidney transplant is a complex surgical procedure that requires lifelong immunosuppression that exposes patients to a risk of opportunistic infections, a lack of readily available organs, and high cost. As such, performing a simultaneous kidney-pancreas transplant has certain indications for the procedure. The general concept is that this surgery can be offered to individuals who have insulin-dependent DM and have subsequently developed ESRD from DN [18]. Specific indications also exist for simultaneous pancreas-kidney transplant in T1D, which include confirmed DN with low/absent C-peptide on insulin treatment, a creatinine clearance of less than 15 mL/minute or the patient being on dialysis, presence of other micro and macrovascular complications of T1D such as cerebrovascular accidents or retinopathy, the ability to withstand immunosuppression and the physiological demands of surgery, and a history of compliance with medical advice and treatment [19]. While these specific indications are recognized as guidelines, each transplant candidate is reviewed by a committee that makes the final decision on whether the patient is placed on a waiting list for transplantation [20].

Contraindications

Absolute contraindications to KT include conditions where the risks outweigh the benefits of the procedure, making transplantation unfeasible. Active systemic infections, such as human immunodeficiency virus and uncontrolled tuberculosis, pose a risk of perioperative morbidity and mortality [21,22]. Patients with active malignancies, particularly those with a high likelihood of recurrence, are also excluded due to concerns about tumor progression under immunosuppression [23]. Severe, uncontrolled metabolic conditions such as cardiovascular disease or advanced liver failure without the prospect of concurrent liver transplantation further preclude candidacy [24]. Conversely, relative contraindications are factors that may increase the complexity of transplantation but do not universally disqualify patients. Examples include advanced age, high body mass index, and significant psychosocial issues, such as non-adherence to medical therapy. These factors necessitate individualized assessment to determine transplant suitability, often requiring multidisciplinary evaluation to optimize outcomes. Addressing modifiable risks and providing patient education can sometimes reclassify relative contraindications to allow transplantation [25,26].

Differences in Operative Techniques and Surgical Challenges

Kidney transplant alone operative time is usually three to four hours while a simultaneous kidney-pancreas transplant takes four to six hours, subsequently, increasing the time for possible intraoperative complications. Donor kidneys can be taken from living or deceased donors, but simultaneous kidney-pancreas is retrieved from the same deceased donor. Partial pancreas harvesting can be performed from living donors; however, it is very rarely done. Kidney transplants alone are usually transplanted in the right iliac fossa for easy access; however, if a pancreas transplant is also being performed, then the left iliac fossa is utilized, as the pancreas is given preference to be placed on the left due to easier access to the iliac vessels. Harvesting of a kidney involves dissection of the kidney as well as the ipsilateral ureter. Harvesting the pancreas requires the removal of the pancreas, duodenum, and spleen. Kidney transplant alone has increased surgical complexity as it requires renal artery, vein, and ureteric anastomosis. A simultaneous kidney-pancreas transplant requires creating a Y stump from the donor’s superior mesenteric and splenic arteries and anastomosing the Y graft, as well as a venous anastomosis, usually the external iliac vein, and duodenal anastomosis to the recipient’s bladder or small bowel (usually a Roux-en-Y configuration) for enteric drainage of the exocrine component of the pancreas [19].

Patient outcomes and complications

Hemorrhage remains one of the leading complications of a kidney transplant, with studies showing that approximately 30% of patients require a blood transfusion [27]. Renal arterial and venous thrombosis is a devastating complication occurring in 1-6% of patients and is recognized as one of the leading factors of kidney allograft loss within the first month [28]. Urological complications as such urinoma formation, acute urinary obstruction, or ureteral anastomotic leak can occur in up to 5% of patients. These complications can usually be managed with percutaneous placement of a ureteral or nephrostomy stent [29]. Any drainage via a percutaneous needle or surgical procedures during the three months following the transplantation surgery is considered a surgical complication. The incidence rate of these complications ranges from 5% to 38%, respectively [30,31]. Several risk factors and chronic illnesses have contributed to the occurrence of complications during the perioperative and postoperative period, including anemia, diabetes, obesity, and cardiac diseases. In addition, the main complications include difficulty in airway and intubation, hematomas, and lymphocele [32]. Incisional hernia, which occurs in 1-18% of kidney transplant patients and is treated with hernioplasty, surgical site wound infection and abscess formation are the most common postoperative complications [30,33]. In a previous study, the baseline characteristics, clinical presentations, and outcomes of post-kidney transplant patients admitted to a diabetic foot unit with those of non-transplant patients during the same timeframe were compared. Among 27 post-kidney transplant patients, 22% underwent amputations compared to just 1.8% in the control group. Despite the expected worse outcomes in post-transplant patients due to immunosuppressive therapy, no significant differences were observed, possibly due to closer monitoring and more frequent clinic visits for the transplant group [34].

A simultaneous pancreas-kidney transplant is associated with increased severity and frequency of complications. Surgical complications occur in up to 37% of patients, as reported in a single-center study. In the simultaneous pancreas-kidney transplant population, the majority of major surgical complications were found to be related to the transplanted pancreas compared to the kidney at 43.6% and 28.2%, respectively [35]. In postoperative results, there was no significant difference in HbA1c levels, tacrolimus levels, and fasting blood glucose levels between T1D and T2D recipients. The most common complications in patients who received a simultaneous pancreas-kidney transplant were asymptomatic para-pancreatic fluid collections (52.5%), superior mesenteric artery thrombosis (12.5%), and surgical site infections. Other causes of failure included technical difficulties, graft thrombosis, graft pancreatitis, and anastomotic leak. In addition, obese recipients had an increased risk of complications. Regarding infection rates, one study from Finland found that in the first year, simultaneous pancreas-kidney transplant patients have a higher rate of hospitalization due to infection. After a five-year period, the risk of rehospitalizations due to infection became similar between the simultaneous pancreas-kidney transplant and kidney transplant alone populations [2,29].

Following KT, infections account for 15-20% of deaths, rendering them one of the most prevalent non-cardiac causes of fatality. The timing of infection after transplantation and patient-specific risk factors, such as diabetes, determine the type of infection encountered [22]. A study conducted by Ozawa et al. showed a strong correlation between diabetes and urinary tract infections among patients who underwent a kidney transplant [36]. Post-transplant weight gain may have a negative impact on patient outcomes. Arrhythmias, pulmonary hypertension, ischemic heart disease, congestive heart failure, and valvular heart disease are also cardiovascular diseases typically seen among kidney transplant recipients [22]. Retinal changes caused by poor diabetes control before transplantation can limit the best possible visual acuity, even after prolonged glycemic normalization following simultaneous pancreas-kidney transplantation. During the first year post-transplant, patients require frequent and thorough ophthalmological examinations due to the risk of diabetic retinopathy progression. Despite these baseline limitations, over half (51%) of simultaneous pancreas-kidney transplant patients achieve a visual acuity greater than 0.6 in at least one eye, sufficient for independent functioning [37]. The incidence of diabetic foot (DF) was notably high, affecting nearly one-third of pancreas and simultaneous pancreas-kidney transplant recipients, with an average onset of 10.7 ± 8 months. Significant factors associated with DF development included poor diabetes control and peripheral arterial disease [38].

Graft Survival and Failure

The recipient’s organ survival and the patient’s overall survival rate are influenced by the presence of comorbidities, most commonly preexisting diabetes or PTDM [39]. The one-year graft survival rate is approximately 95%, but this rate gradually declines over time, reaching 87.5% at the five-year mark, with the one-year survival being similar among individuals with T1D and T2D [32,40,41]. In addition, improvements in organ retrieval and preservation techniques, surgical methods, the use of prophylactic antibiotics, and tailored immunosuppression and monitoring protocols have enhanced one-year graft survival outcomes, particularly in the elderly and individuals with comorbid diseases [22,40]. Even though the long-term graft survival rate has been steady and improving, a significant percentage of kidney transplant patients experience graft failure, which is defined by nephrectomy and the initiation of dialysis [41]. The pattern of graft failure or rejection in patients with T1D is similar to that in individuals with T2D. In addition, the definitive method for diagnosing graft failure is a renal biopsy, with changes in serum creatinine levels determining whether a biopsy is needed [42]. Patients in the post-transplant period who experience surgical complications have a decreased graft survival rate, with graft failure often necessitating dialysis or re-transplantation, leading to an increased mortality rate [30,40]. Another cause for graft rejection and failure is the antibody-mediated reaction associated with non-human leukocyte antigen mismatches or allo-immunity between the donor and recipient [22,41]. Moreover, there is a 30% increased risk of graft failure among smokers with concurrent diabetes [43].

Mortality Rates

Despite advancements in the management of diabetic patients with ESRD, the mortality rate of patients with diabetes who underwent kidney transplants remains significantly higher than that of non-diabetic patients. With an increased risk of graft failure, the mortality rate among patients readmitted to the hospital also increased from 50% to 75% [44]. On average, the life expectancy after a kidney transplant is 18.3 years in T1D patients and 8-19 years in T2D patients. Five-year survival possibility among TID patients is 85% and among T2D patients is 77% compared to 88% among non-diabetics [45]. Patients with DM have a decreased five-year survival rate compared to non-diabetic patients at 70% and 93%, respectively. Cardiovascular complications were the most common cause of death among the diabetic patient cohort [46]. A study reported that 10-year survival increased from 90.0% among diabetics to 95.3% among non-diabetics. Whereas graft survival was around 83% among diabetics compared to 90% among non-diabetics [47] (Table 1).

**Table 1:** Overview of graft and patient survival rates.

Graft survival rate (%)				Survival rate (%)			
	Diabetic		Non-diabetic		Diabetic		Non-diabetic
	Type 1 diabetes	Type 2 diabetes			Type 1 diabetes	Type 2 diabetes	
One year [41,40]	96.3	95.7	92.3	One year [41,40]	96.9	97.9	91.3
Five years [41,40]	97.5	87.5	81.2	Five years [45]	85	77	88

New-onset diabetes mellitus after transplantation

NODAT or PTDM develops in solid organ transplant recipients, with a higher incidence associated with KT [48,49]. Approximately 10% to 20% of KT patients develop NODAT in the first year and 25-35% in the third year after transplant [50]. These individuals are predisposed to certain risk factors that need early identification and prompt management to alleviate complications, including graft failure, infections, cardiovascular morbidity, and mortality [51]. A two-hit hypothesis is in play in these patients where non-modifiable along with modifiable risk factors, such as nutritional deficiencies, infections, and drugs, contribute to its development [52]. Table 2 emphasizes the crucial role of these risk factors during different phases of renal transplant.

**Table 2:** Risk factors for new-onset diabetes mellitus after transplant.

	Non-modifiable risk factors	Modifiable risk factors
Pre-transplant [16,53-57]	Single nucleotide polymorphisms (HNA-4F gene), age >45 years, male gender, black ethnicity, polycystic kidney disease, and a family history of diabetes	Body mass index > 30 kg/m^2^
Peri-transplant [56,57]	Cadaveric renal allograft	-
Post-transplant [58-61]	-	Immunosuppression, hepatitis C virus, cytomegalovirus, hypomagnesemia (<0.74 mmol/L), vitamin D deficiency (<30 ng/mL), and allograft rejection

Transplant recipients undergo significant stress during and after the procedure which creates a chronic low-grade inflammatory state affecting the beta-cell function [59]. Immunosuppressive drugs counteract this inflammatory state but play a major role in the development of post-transplant hyperglycemia. Tacrolimus causes activation of bone morphogenetic protein/suppressor of mothers against decapentaplegic pathway, resulting in impaired beta-cell function [62]. High-dose steroids usually given in the induction phase of post-transplant immunosuppressive therapy decrease insulin sensitivity and increase gluconeogenesis in the liver causing impaired glucose tolerance and leading to hyperglycemia [59]. MTOR inhibitors such as sirolimus also attenuate insulin secretion and impair the signal transmission of insulin receptors [51]. Other risk factors such as hepatitis C, obesity, male gender, and age significantly lower insulin sensitivity [16]. Cytomegalovirus infection is hypothesized to cause a pro-inflammatory state and destruction of beta cells [59]. Thus, a complex interaction between these elements propagates a cascade of pro-inflammatory and hyperglycemic states leading to NODAT. The American Diabetes Association and WHO established a clear diagnostic criterion for NODAT in 2003 (Table 3) [63]. However, NODAT may be undetected in KT patients due to high anemia rates and dependence on HbA1c for diabetes diagnosis [64,65].

**Table 3:** Diagnostic criteria to identify new-onset diabetes after transplant.

Blood tests [63-65]	Results
Fasting blood glucose levels	≥126 mg/dL (7 mmol/L) on >1 occasion
Random blood glucose levels	≥200 mg/dL (11.1 mmol/L) with symptoms
Two-hour blood glucose levels after a 75 g oral glucose tolerance test (OGTT)	≥200 mg/dL (11.1 mmol/L)
Hemoglobin A1c levels	>6.5%

Screening for NODAT is essential due to its higher incidence and hazardous consequences on the health of KT patients. It is recommended that every patient should be tested for DM at three, six, and twelve months post-transplant. Treatment for NODAT has been usually guided by the same criteria used for non-transplant patients with T2D [63]. Preventive strategies to reduce the occurrence of NODAT have impactful results in reducing healthcare costs and improving renal transplant patient’s quality as well as quantity of life. Lifestyle modifications, including dietitian referral, exercise program, and weight loss advice, are beneficial before and after KT for non-diabetic as well as diabetic patients [16].

Obese patients benefit from bariatric surgery because it strengthens their access to transplants by reducing weight and improving pretransplant health status. Procedures such as sleeve gastrectomy decrease the prevalence of diabetes in CKD patients [66]. NODAT-preventive measures also include assessing the use of newer immunosuppressant medications with a lesser impact on glucose metabolism. Contemplating new combinations of existing medications, including changing the maximum doses and timings for dosage adjustment, reduces their impact on glucose intolerance. These preventive strategies also incorporate the protection of beta-cell islets by early initiation of insulin treatment. Use of dipeptidyl peptidase 4 inhibitors (DPP-4i) which prevent chronic inflammation, a precursor for NODAT, individualization of immunosuppressive medication usage (cyclosporine vs. tacrolimus vs. belatacept use), and consideration of steroid-sparing protocols are important and impactful NODAT-preventive strategies [67]. Early vitamin D supplementation may reduce the risk of diabetes onset in kidney transplant recipients [68].

Management

Lifestyle Modifications

Lifestyle modifications, in the form of physical exercise, weight loss, and dietary restrictions, are the principal interventions when trying to manage DM. This is especially the case in kidney transplant recipients. Physical exercise in the form of resistance training and aerobic exercise has been shown to lower fasting plasma glucose and HbA1c levels in kidney transplant recipients. Physical exercise in combination with weight loss causes beta-cell function to improve in patients with T2D. Coupled with physical exercise, dietary restrictions contribute to improved glycemic control. Studies assessing various diets showed that Mediterranean, vegetarian, vegan, and macrobiotic diets reduced weight, HbA1c levels, and insulin sensitivity. It is paramount that kidney transplant recipients are encouraged to adhere to these lifestyle modifications for improved outcomes [69,70].

Glucose-Lowering Medications

Insulin degludec is considered a safe and effective modality among renal transplant patients with comorbid T2D. Persistent glycemic control can be achieved with once-daily administration of degludec during the post-transplant duration leading to improved quality of life among patients. Studies have reported enhanced graft survival among patients receiving degludec [71]. Clearance of metformin occurs in the kidneys, which raises safety concerns about its use in diabetic kidney transplant recipients. However, studies have revealed that patients taking metformin after KT had no serious adverse events and that taking metformin in the first year post-transplantation may be safe [72,73]. Thiazolidinediones in combination with insulin have been shown to significantly decrease HbA1c and inflammatory marker levels in kidney transplant recipientss but no improvement in decreasing fasting blood glucose compared to insulin therapy alone. However, thiazolidinediones have been reported to cause fluid retention, manifesting as peripheral edema of varying severity from mild to moderate [74,75]. Several studies have shown that DPP-4i cause a reduction in Hb1AC levels and fasting plasma glucose. Additionally, these studies reported negligible side effects and transplant-specific adverse effects. Sitagliptin has a low incidence of hypoglycemia and does not alter immunosuppressant levels making it an ideal choice for kidney transplant recipients. An important consideration is that DPP-4i clearance is via the kidneys, except for linagliptin which is cleared by biliary excretion. This requires monitoring and dose adjustments of DPP-4i based on renal function in KTRs [76-79]. Glucagon-like peptide-1 receptor agonists (GLP-1RA) are considered safe and effective in managing diabetes among kidney transplant recipients. Low risk of hypoglycemia, reduced need for insulin, and extremely rare probability of acute rejection have been reported with GLP-1RA [80]. Sodium-glucose cotransporter 2 (SGLT2) inhibitors have been shown to be effective with minimal adverse effects in managing blood glucose levels among NODAT patients. The only notable side effect was some weight loss [81,82]. However, studies have shown that SGLT2 inhibitors demonstrate nephroprotective properties with reduced incidences of acute kidney injury compared to placebo and DPP-4i [83]. DPP4i administration showed improved clinical outcomes among patients who underwent pancreas transplantation [84]. Nevertheless, the role of glucose-lowering medication in the management of pre-existing and post-transplant is debatable. The largest systemic review analyzing the safety and efficacy of medications, including insulin, DPP4i, SGLT2 inhibitor,s and glitazones, reflected that the majority of the evidence is of low to very low confidence. Larger and greater quality randomized controlled trials are needed to draw definitive conclusions [85] (Table 4).

**Table 4:** Summary of studies using glucose-lowering drugs post-transplant. DM: diabetes mellitus; HbA1c: glycosylated hemoglobin; FPG: fasting plasma glucose; PPPG: postprandial plasma glucose; IGT: impaired glucose tolerance; CRP: C-reactive protein; ESR: erythrocyte sedimentation rate; NODAT: new-onset diabetes after transplant; GLPRA1: glucose-like peptide receptor agonists-1; SGLT2: sodium-glucose cotransporter 2

Author (year)	Type of diabetes	Glucose-lowering medication	Number of subjects (n)	Follow-up duration	Comments on patient outcomes
Sanyal et al. (2021) [71]	Type 2 DM	Insulin degludec	61	3 months	Substantial reduction in serum HbA1c, FPG, and PPPG levels. No severe episode of hypoglycemia was reported. Degludec was rendered safe and effective
Alnasrallah et al. (2019) [72]	IGT	Metformin	78	12 months	Tolerance and effectiveness were comparable between the patients receiving metformin and standard care. No severe complications were reported
Vest et al. (2018) [73]	Type 2 DM	Metformin	14,144 (4.7% received metformin)	1 year	Significantly lower all-cause and infection-related mortality among the metformin group compared to other anti-diabetic medications. No increased rate of graft failure and acute rejection was observed
Kharazmkia et al. (2014) [74]	Type 2 DM	Pioglitazone + insulin	62	4 months	Considerable reduction in HbA1c levels, CRP, ESR, and daily insulin need. Improvement in the lipid profile.
Arashnia et al. (2015) [75]	Type 2 DM	Pioglitazone + insulin	58	1 month	CRP and cholesterol levels significantly decreased
Sanyal et al. (2013) [77]	NODAT	Linagliptin	21	24 weeks	HbA1c, FPG, and PPPG levels decreased. Minimal weight gain was recorded, and only a single episode of hypoglycemia was reported
Soliman et al. (2013) [78]	NODAT	Sitagliptin and insulin glargine	Sitagliptin = 28, glargine = 17	12 weeks	Glycemic control was similar between the two groups. Slight weight reduction was observed in the sitagliptin group
Boerner et al. (2014) [79]	NODAT	Sitagliptin	22	12 months.	Renal function and immunosuppressant levels remained unchanged
Kukla et al. (2020) [80]	Type 2 DM	GLPRA1	171	12 months	A decreased need for insulin and risk of hypoglycemia after 12 months was achieved. Kidney function was stable. Four patients reported side effects, including pancreatitis, nausea, diarrhea, and fatigue
Halden et al. (2019) [81]	NODAT	SGLT2 inhibitor	Empagliflozin = 22, placebo = 22	24 weeks	A significant reduction in HbA1c levels and body weight was observed in the SGLT2 inhibitor group

Immunosuppression

Immunosuppression among kidney transplant recipients remains a significant area of debate. Certain immunosuppression medications have been postulated to be linked to the emergence of PTDM. Tacrolimus has shown to be most diabetogenic leading to NODAT in comparison to other immunosuppressants. A study revealed that the use of cyclosporine had a lower incidence of PTDM compared to tacrolimus in the first year but also enhanced the probability of acute rejection [86]. One study reflected that a change in immunosuppressive medication from tacrolimus to cyclosporine resulted in a reversal of NODAT [87]. Belatacept administration was also associated with a lower incidence of NODAT [88]. The risk of developing PTDM is higher among patients receiving sirolimus compared to mycophenolate mofetil [89]. Avoidance of corticosteroids in KT is controversial. The postulated contribution of corticosteroids in developing hyperglycemia and NODAT is not backed by evidence. A meta-analysis of studies analyzing the impact of corticosteroids on the emergence of PTDM after three and six months did not reveal causation [90,91].

## Conclusions

Renal transplantation continues to be a lifesaving intervention for patients with diabetes and associated kidney disease by improving both their quality of life and survival rate. KT can be performed alone or simultaneously with a pancreas transplant. Despite the significant advancements in surgical techniques, immunosuppressive drug therapies, and perioperative patient management, substantial challenges and complications persist, including graft failure/rejection, infection, and the emergence of PTDM that impact patient outcomes. Future research should focus on optimizing patient selection criteria, developing individualized immunosuppressive regimens, and establishing guidelines regarding the management of diabetes among kidney transplant recipients to enhance their quality of life and outcomes.
